# Maintenance of Antibody Response in Egyptian Healthcare Workers Vaccinated with ChAdOx1 nCoV-19 Vaccine during Delta and Omicron Variants Pandemic: A Prospective Study

**DOI:** 10.3390/vaccines10101706

**Published:** 2022-10-12

**Authors:** Noha M. Hammad, Heba M. Kadry, Mai M. Malek, Shereen Mohamed Bahgat, Noha M. Abdelsalam, Amira Hamed Mohamed Afifi, Doaa Alhussein Abo-alella

**Affiliations:** 1 Department of Medical Microbiology and Immunology, Faculty of Medicine, Zagazig University, Zagazig 44519, Egypt; 2 Viral Infection Working Group of International Society of Antimicrobial Chemotherapy (VIWG/ISAC), England and Wales, UK; 3 Department of Family Medicine, Faculty of Medicine, Zagazig University, Zagazig 44519, Egypt; 4 Department of Community Medicine, Faculty of Medicine, Zagazig University, Zagazig 44519, Egypt; 5 Department of Clinical Pathology, Faculty of Medicine, Zagazig University, Zagazig 44519, Egypt

**Keywords:** COVID-19 infection, Delta variant, Egypt, healthcare workers, Omicron variant, Oxford–AstraZeneca vaccine

## Abstract

Background: The severe acute respiratory syndrome coronavirus 2 (SARS-CoV-2) is a constantly evolving virus, resulting in an increased burden on the existing COVID-19 vaccines. Healthcare workers (HCWs) are the first line of defense against the coronavirus disease 2019 (COVID-19) pandemic and have been prioritized among the risk categories receiving the COVID-19 vaccine. This work aimed to investigate the maintenance of antibody response of the Oxford–AstraZeneca vaccine (ChAdOx1/nCoV-19). Methods: Anti-spike immunoglobulin G (IgG) was measured at baseline point (immediately prior to vaccination) and 12- and 24-week (w) points following vaccination. Adverse reactions to the vaccine were reported. Participants were followed up for the incidence of COVID-19 during the 12 w interval between vaccination doses for 24 w after the second dose. Results: A total of 255 HCWs participated in the study. Prior to vaccination, 54.1% experienced COVID-19, 88.2% were seropositive after the first dose, while seropositivity reached 95.7% after the second dose. Following the first and second doses, the anti-spike IgG serum level was significantly higher in subjects with past COVID-19 than in others (*p* < 0.001 and =0.001, respectively). Conclusions: The Oxford–AstraZeneca vaccine is generally safe and provides a highly effective long-term humoral immune response against the Delta and Omicron variants of SARS-CoV-2.

## 1. Introduction

The COVID-19 pandemic has been caused by the novel severe acute respiratory syndrome coronavirus 2 (SARS-CoV-2). SARS-CoV-2 infection ranges from asymptomatic to life-threatening sepsis. It may cause a broad spectrum of symptoms, including multiple body systems [1,2]. COVID-19 infection is a potentially preventable disease. There has been a positive correlation between the intensity of public health orientation with infection control measures application and transmission control [2]. Nevertheless, vaccination was the first recommendation of the Centers for Disease Control and Prevention (CDC) to reduce the spread of COVID-19 and the health crisis [3].

HCWs are considered the front line in confronting the COVID-19 infection [4]. Therefore, despite using standard and transmission-based precautions in health facilities to limit the spread of SARS-CoV-2, HCWs are constantly at risk of COVID-19 infection [5]. Furthermore, between January 2020 and May 2021, the World Health Organization (WHO) has estimated HCW deaths to range between 80,000 and 180,000, a staggering number that necessitates immediate action to protect HCWs worldwide [6]. Vaccination, testing of symptomatic cases, and vetting HCWs are the pillars of health system protection. In accordance with the WHO roadmap for prioritizing COVID-19 vaccination [7], the Egyptian Ministry of Health and Population has prioritized HCWs for the COVID-19 vaccine among high-risk categories.

Oxford–AstraZeneca vaccine, Covishield, and Vaxzevira are the used brand names for the ChAdOx1 nCoV-19 (recombinant) vaccine, which has conditional authorization in the UK for the prevention of COVID-19 [8]. Later, by the end of November 2021, more than 170 countries had approved and authorized the Oxford–AstraZeneca vaccine for emergency use [9]. The vaccine employs a unique approach. A chimpanzee adenovirus vector (ChAdOx) encodes SARS-CoV-2 spike (S) glycoprotein that induces an immune response when expressed [10]. In terms of resource optimization, it outperforms Pfizer/BioNTech and Moderna’s mRNA-based COVID-19 vaccines. Not only is it less expensive, but it also does not require the same cold-chain management as mRNA-based vaccines [11]. The AstraZeneca vaccine requires two standard doses (0.5 mL each) administered at intervals ranging from 4 to 12 weeks (w). Based on phase-3 trials, the two doses of the Oxford–AstraZeneca vaccine effectively prevent COVID-19 infections [12]. Additionally, they have averted hundreds of hospitalizations and fatalities, even when used against the more contagious COVID-19 Delta variant [10]. Antibody testing is indispensable for understanding the dissemination of the SARS-CoV-2 virus in the population. It is essential to comprehend the response to emerging variants to detect differences in the degree of immunity and their durability. By inducing neutralizing antibodies, the majority of COVID-19 vaccines demonstrate an acceptable safety profile behind their use in the real world [13,14]. However, vaccination and SARS-CoV-2 antibody testing are not routine clinical practices. Therefore, additional research is urgently needed to determine the vaccine’s efficacy and establish future immunization guidelines [13,14].

The current work aimed to investigate the efficacy and safety of the Oxford/AstraZeneca COVID-19 vaccine in preventing COVID-19 infections among Egyptian HCWs, despite the ongoing evolution of the virus, in addition to analyzing the implications of the vaccine on the Omicron variant era.

## 2. Materials and Methods

### 2.1. Participants

**Figure 1:**
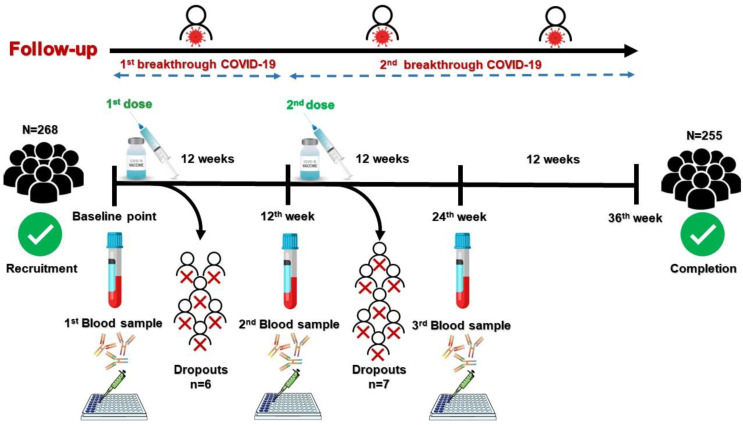
A schematic illustration of study design and flow. At the baseline point, healthcare workers (HCWs) were recruited and assessed for eligibility criteria. Medical history was taken, and baseline blood samples were withdrawn from 268 HCWs who were eligible for participation. Participant HCWs then received the 1st dose of vaccination. Participants were followed up for 12 weeks. Adverse reactions to the 1st dose of the vaccine, the incidence of breakthrough COVID-19 infection, and dropouts were reported during the first 12-week follow-up period. At the 12th-week time point, a 2nd blood sample was withdrawn before the participants received the 2nd dose of the vaccine. Participants were followed up for another 12 weeks. Adverse reactions to the 2nd dose of vaccine, the incidence of breakthrough COVID-19 infection, and dropouts were reported during the second 12-week follow-up period. At the 24th-week time point, a 3rd blood sample was withdrawn. Participants were followed up for another 12 weeks. Incidence of breakthrough COVID-19 infection and dropouts were reported during the third 12-week follow-up period. At the 36th week time point, 255 HCWs completed the study. Serum levels of anti-spike IgG antibody were measured from withdrawn blood samples by enzyme-linked immunosorbent assay. All dropouts were excluded from the analysis.

The present study was implemented at the Immunology Research Laboratory, Medical Microbiology and Immunology Department, Zagazig Faculty of Medicine, Egypt. The study included 268 HCWs who attended to the Infection Control Unit of ZUHs to receive the Oxford/AstraZeneca COVID-19 vaccine during the period from June 2021 to March 2022. All participants underwent a screening interview with a detailed clinical history including past COVID-19 infections; however, they were not screened for SARS-CoV-2 nucleocapsid antigen in their serum. Participants received two standard doses of Oxford/AstraZeneca COVID-19 vaccine (5 × 10^10^ viral particles) at the 12-week interval [12]. Figure 1 displays a schematic illustration of study design and flow.

Exclusion criteria for participation were HCWs refusal, pregnancy, lactation, and COVID-19 vaccination contraindications (current COVID-19, any acute illness, history of allergy to vaccine components, past administration of COVID-19 vaccine).

### 2.2. Blood Sampling

At baseline (before vaccination), 12 and 24 w, two mL of peripheral blood were collected from each participant via venous puncture. Serum was separated by centrifugation at 3000 RPM and stored at −20 °C.

### 2.3. Measurement of Anti-Spike IgG Serum Level

The serum level of IgG antibody against spike (S) of SARS-CoV-2 was determined at baseline, 12-, and 24-week time-points using indirect enzyme-linked immunosorbent assay (ELISA) according to the manufacturer’s instructions (Anti-SARS-CoV-2 QuantiVac ELISA (IgG), EUROIIMMUN, Lübec, Germany, REF# EI 2606-9601-10 G). The ELISA kit’s reagent well coat was recombinant S1 domain of the spike protein of SARS-CoV-2, which was recombinantly expressed in the human cell line HEK 293. Serum samples with anti-spike IgG concentration ≥ 35.2 IU/mL were considered immunoreactive.

### 2.4. Follow-Up of Participants

After each vaccination dose, HCWs were monitored, and all adverse reactions (ARs) ensuing were carefully reported. The Google form application was used to generate an online questionnaire to collect data, and the link was sent to all participants. After completing the vaccine schedule, participants were followed up for a further six months. Any incidence of breakthrough COVID-19 infection was reported during the study period, and all COVID-19 infections were diagnosed clinically and confirmed by laboratory investigations, chest imaging, and nucleic acid testing as described before [15]. At the conclusion of the study, all dropout subjects were excluded from statistical analysis (Figure 1).

### 2.5. Statistical Analysis

Numerical data were presented as mean ± standard deviation (SD) and median (min-max). Categorical data were expressed as frequency and percentage. Pearson chi-square (χ^2^) and Fisher’s exact tests were utilized to determine the association between immunoreactivity to the vaccine and sociodemographic and clinical variables. McNemar’s test was used to analyze the ARs to the vaccine. The Wilcoxon signed ranks test was used to compare the median difference between baseline, 12 w, and 24 w anti-spike IgG serum antibody levels. The Mann–Whitney U test was used to compare the median difference between anti-spike IgG serum levels at 12 and 24 w prior to vaccination COVID-19 infected and never-infected vaccinated groups. Spearman’s correlation coefficient (r) was used to determine the degree and direction of association between numerical variables. *p*-values ≤0.05 are considered significant. Statistical analyses were performed using SPSS software (version 24; IBM Corp., Chicago, IL, USA).

## 3. Results

### 3.1. Baseline Characteristics of the Studied HCWs

Two hundred and sixty-eight HCWs in Zagazig University Hospitals participated in the present study. Of the 268 HCWs enrolled in the study, 13 were excluded. Nine had personal reasons, one got pregnant, and three did not complete the vaccination schedule due to current or recent COVID-19 infection. Therefore, 255 participants fulfilled the requirements of the study. More than half of the participants (57.6%) were between 20 and 39 years old. Females were more prevalent (56.5%). Among the HCWs, 71.8% were medical personnel, and 54.1% informed history of symptomatic COVID-19 infection before vaccination. Nearly half of the participants (54.1%) took ivermectin for prophylactic and therapeutic purposes, and over half of the participants (54.1%) experienced a previous COVID-19 infection before vaccination. The baseline characteristics of the studied HCWs are illustrated in Table 1.

**Figure 2:**
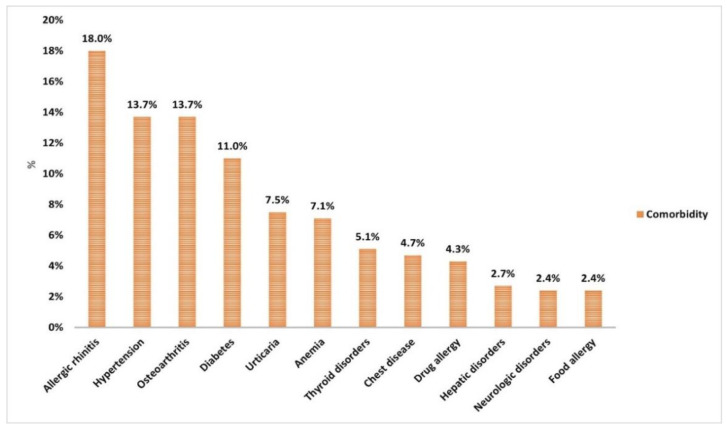
Associated comorbidities in descending order of frequency among study participants (*N* = 255).

Approximately half of the participants (51.8%) had associated comorbidities (Table 1). The most frequent associated comorbidities were allergic rhinitis (18%), hypertension (13.7%), osteoarthritis (13.7%), and diabetes (11%) (Figure 2). In addition, 22.4% of participants were on regular medication (Table 1).

### 3.2. Adverse Reactions to the Vaccine

**Figure 3:**
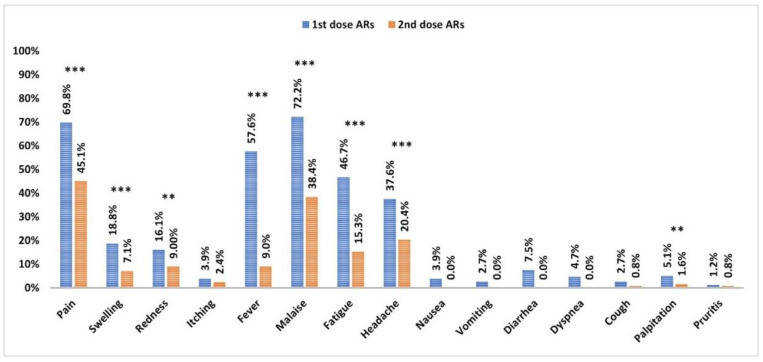
Frequency of local and systemic ARs to vaccine after administration of the 1st and 2nd doses. Data were analyzed by McNemar Test, and significant differences were defined as *** *p* < 0.001 and ** *p* < 0.01. AR; adverse reactions.

Local and systemic ARs were more prevalent in HCWs after the first vaccination dose compared to those after the second dose (*p* < 0.001 and <0.001, respectively). The most frequent local ARs were pain, swelling, and redness (*p* < 0.001, <0.03, and <0.001, respectively). Fever, malaise, fatigue, headache, and palpitation were the most significant systemic ARs (*p* < 0.001, <0.001, <0.001, <0.001, and <0.01, respectively) (Figure 3).

The experiencing of local and systemic ARs after two vaccination doses was indirectly correlated with age (*p* < 0.01, 0.01, 0.001, and 0.2, respectively). Females had a higher risk of developing local and systemic ARs than males after the first dose (*p* < 0.001 and <0.005, respectively) and second vaccination doses (*p* < 0.001 and =0.7, respectively). The presence of associated comorbidities was not correlated with local and systemic ARs occurring after the first (*p* = 0.6 and 0.5, respectively) and second vaccination doses (*p* = 0.04 and 0.9, respectively) (Table 2).

### 3.3. Anti-Spike IgG Serum Level and Immunoreactivity to Vaccine

**Figure 4:**
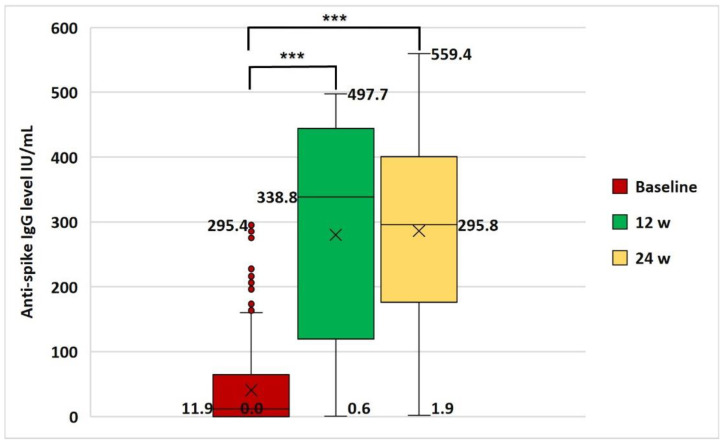
Anti-spike IgG antibody serum level (median and range) at baseline, 12 w, and 24 w time points of the study. Data were analyzed by Wilcoxon Signed Ranks Test and significant difference was defined as *** *p* < 0.001. w, week; ×, mean.

The baseline anti-spike IgG serum level was detectable in 35.6% of HCWs (mean: 40.5 ± 59.2 IU/mL). After the 1st vaccination dose, 88.2% of the studied HCWs were seropositive (mean: 280.3 ± 169.0 IU/mL), while after the 2nd vaccination dose seropositivity reached 95.7% (mean: 286.2 ± 130.7 IU/mL) (Table 3 and Figure 4).

Compared to the baseline level, the anti-spike IgG serum level was significantly elevated at 12 and 24 w (*p* < 0.001 and <0.001, respectively). There was no significant variation in anti-spike IgG serum level between 12 w and 24 w (*p* = 0.5) (Figure 4). Among the baseline non-reactive HCWs (64.8%), the seroconversion rates after the first and second vaccination doses were 81.8% and 93.3%, respectively (Table 3)**.**

**Figure 5:**
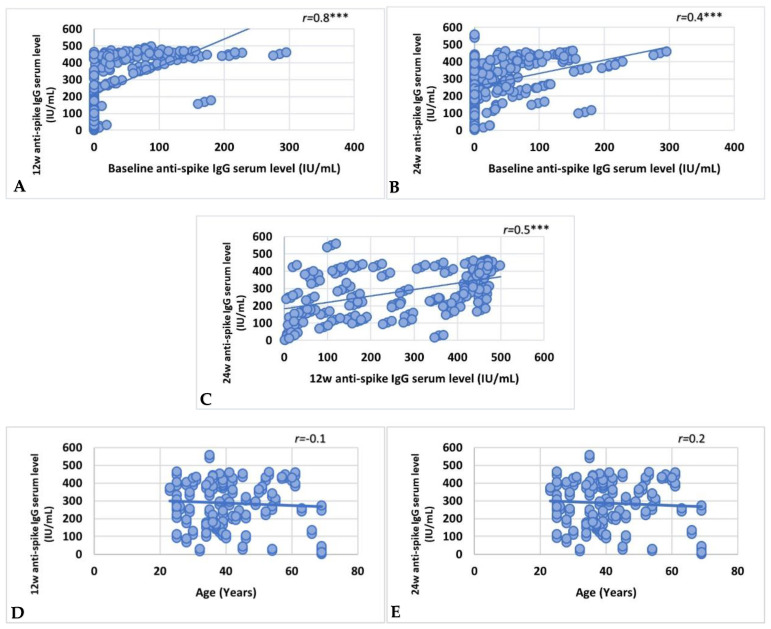
Correlations between (**A**) baseline and 12 w, (**B**) baseline and 24 w, (**C**) 12 w and 24 w anti-spike IgG serum levels. Correlations between age and (**D**) 12 w and (**E**) 24 w anti-spike IgG serum levels. Correlations were measured by Spearman’s rank correlation coefficient (r) and significant difference was defined as *** *p* < 0.001. w, week.

The baseline anti-spike IgG serum level was significantly and directly correlated with antibody serum level after the first and second vaccination doses (r = 0.8, *p* < 0.001 and r = 0.4 and *p* < 0.001, respectively) (Figure 5A,B). A significant direct correlation was detected between 12 w and 24 w anti-spike IgG serum levels (r = 0.5, *p* < 0.001) (Figure 5C).

### 3.4. Factors Affecting Vaccine Immunoreactivity

Although a non-significant correlation was found between age and anti-spike IgG serum levels after the first and second vaccination doses (Figure 5D,E) (r = −0.01, *p* = 0.8 and r = 0.02, and *p* = 0.8, respectively), the immunoreactivity to vaccination was significantly correlated with younger age group (*p* = 0.009) (Table 4). Sex, HCWs, ivermectin administration, and associated comorbidities (liver disease, neurologic and thyroid disorders, allergic rhinitis, urticaria, and drug and food allergy) had no significant effect on immunoreactivity. However, the presence of diabetes, hypertension, and osteoarthritis was significantly associated with non-immunoreactivity to vaccination (*p* < 0.001, =0.049, and <0.001, respectively) (Table 4).

Local and systemic ARs following the first vaccine dose were significant indicators of vaccine immunoreactivity (*p* = 0.01 and <0.001, respectively). Pain, fever, malaise, and fatigue were the most significant indicators of immunoreactivity after the first dose (*p* = 0.01, 0.001, 0.01, and 0.002, respectively). Although ARs were non-significantly associated with immunoreactivity after the second dose (*p* = 0.6 and 0.1, respectively), the odds of immunoreactivity are increased by approximately three folds (OR: 2.9, CI: 0.75–11.17) with experiencing systemic ARs (Table 5).

### 3.5. COVID-19 Infection and Impact on Anti-Spike IgG Serum Level

**Figure 6:**
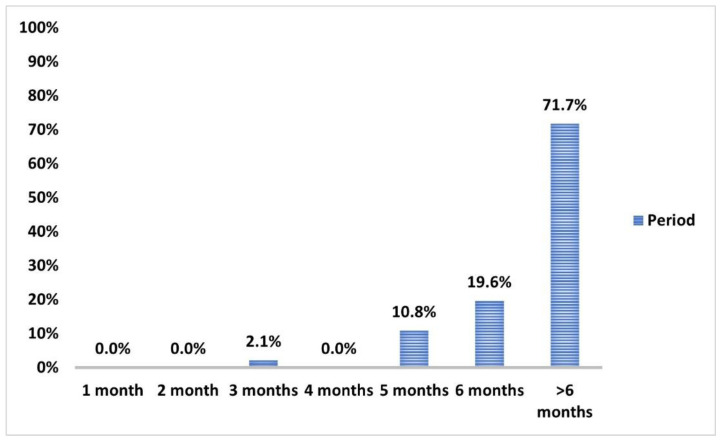
Frequency of time lapse periods from past COVID-19 infection to baseline point of the study among those who experienced infection prior to vaccination (*n* = 138).

More than two-thirds experienced past COVID-19 at least six months earlier (Figure 6). Non-immunoreactivity at baseline was significantly observed in 80.2% of subjects with a negative infection history (*p* < 0.001) (Table 6).

**Figure 7:**
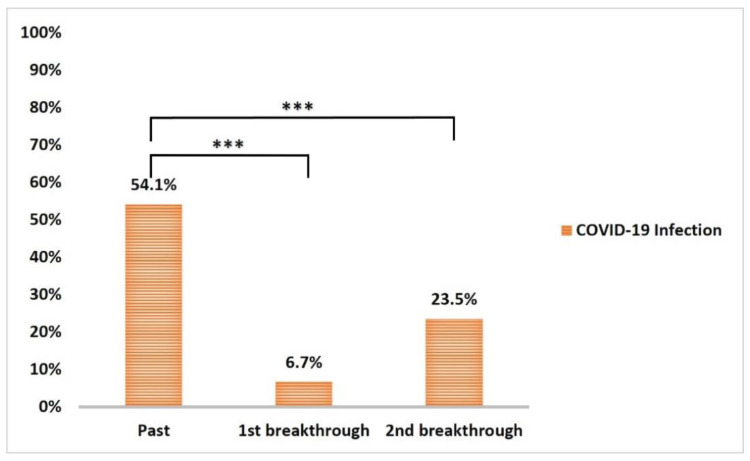
Frequency of past COVID-19 infection (prior to vaccination), 1st breakthrough COVID-19 infection after the 1st dose (between two doses of vaccination), and 2nd breakthrough COVID-19 infection after the 2nd dose of vaccine. Data were analyzed by McNemar Test, and significant difference is defined as *** *p* < 0.001.

**Figure 8:**
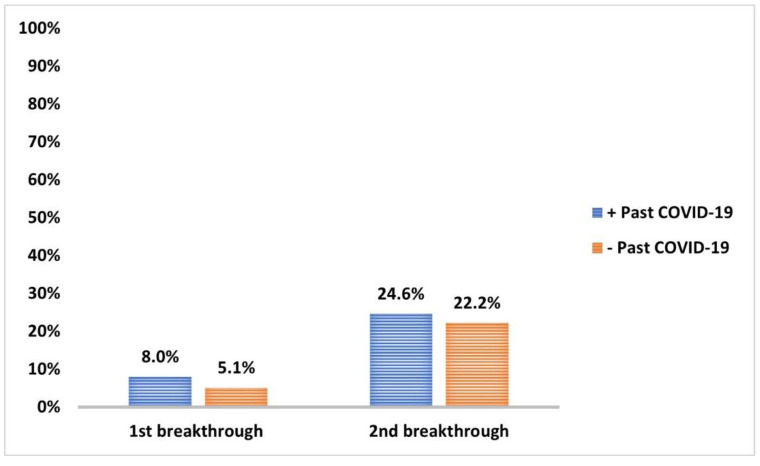
Frequency of 1st breakthrough and 2nd breakthrough COVID-19 infections in those who experienced past COVID-19 and those who did not. Data were analyzed by chi-square test and *p* = 0.6.

The incidence of breakthrough COVID-19 infection within 12 w from the first vaccination dose (6.4%) and during the follow-up period after second dose (23.5%) significantly decreased (*p* < 0.001 and <0.001, respectively) compared to past COVID-19 infections (Figure 7). None of the reported breakthrough COVID-19 cases during the follow-up periods required hospital isolation. However, the incidence of first and second breakthrough infections in those with past COVID-19 infection (8% and 24.6%, respectively) compared to those without (5.1% and 22.2%, respectively) was non-significant (*p* = 0.5) (Figure 8).

**Figure 9:**
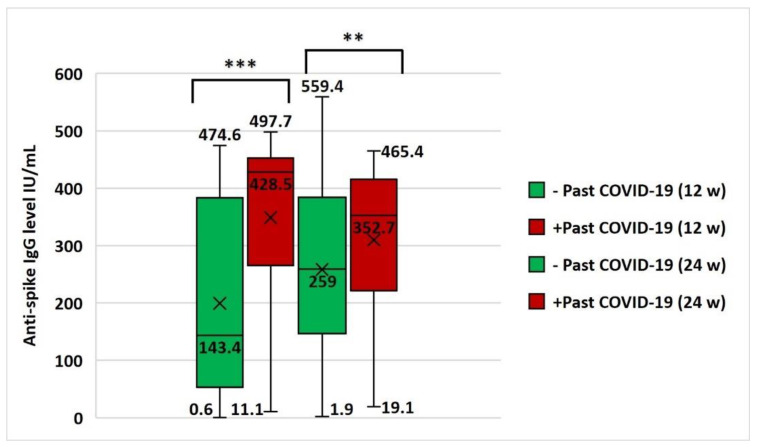
Effect of past COVID-19 infection on antibody immune response after vaccination. Comparison of anti-spike IgG antibody serum level (median and range) between negative (−) and positive (+) past COVID-19 infection groups at 12 w and 24 w. Data were analyzed by Mann–Whitney U test and significant differences were defined as *** *p* < 0.001 and ** *p* < 0.01. w, week; ×, mean.

In subjects with past COVID-19 infection, anti-spike IgG serum level was significantly higher after the first dose (mean: 348.8 ± 140.5 IU/mL) and second dose (mean: 309.8 ± 120.9 IU/mL) of vaccination when compared to level after the first (mean: 199.5 ± 164.4 IU/mL) and second dose (mean: 258.5 ± 136. IU/mL) in those who gave a negative history of past infection (*p* < 0.001 and =0.001, respectively) (Figure 9).

## 4. Discussion

Declaring the COVID-19 pandemic as a global public health crisis by the WHO on 11 March 2020 [16] has resulted in accelerated developing and licensing of vaccines against the emerging virus. A thorough benefit–risk assessment requires further evaluation of these vaccines’ safety and efficacy in different geographic, demographic, and ethnic populations. The vaccines proposed were ChAdOx1 (AstraZeneca/Oxford), BNT162b2 (Pfizer/BioNTech), JNJ-78436735 (Janssen), mRNA-1273 (Moderna), and NVX-CoV2373 (Novavax). mRNA vaccines, such as BNT162b2 and mRNA-1273, demonstrated an efficacy of 95% and are currently used in several countries [17,18]. Although the effectiveness of viral vector vaccines (ChAdOx1 and JNJ-78436735) was documented to have diminished efficacy compared to mRNA vaccines, they satisfied the minimum efficacy criteria of the WHO. They were used for early-stage vaccination in 2021 [19].

In the present work, we investigated the long-term efficacy of the Oxford–AstraZeneca (ChAdOx1 nCoV-19) vaccine in preventing COVID-19 infection in 255 Egyptian HCWs, who fulfilled the requirements of the study during the 12 w interval between the two vaccination doses and throughout the six-month follow-up period after the second vaccination dose. In addition, we correlated the serum level of anti-spike IgG and immunoreactivity to vaccination with sociodemographic characteristics, clinical variables, and ARs to the vaccine. In the present study, females (56.5%) and those aged 20 to 39 years (57.6%) were predominant. However, paramedical HCWs represented less than one-third (28.2%).

Following that, several cases of thrombotic events accompanied by thrombocytopenia have been observed and linked to the administration of the Oxford–AstraZeneca vaccine; Denmark, pursued by several European countries, was the first country to suspend the use of the Oxford–AstraZeneca vaccine [20]. Nevertheless, research data could not recognize platelet dysfunction in Oxford–AstraZeneca vaccinated population [21,22]. Therefore, the WHO and the European Medicines Agency declared that the trend of hypercoagulability could not be vindicated and recommended continued vaccination by the Oxford–AstraZeneca vaccine [23].

The ARs reported in the current study align with those usually experienced following any vaccine, including different COVID-19 vaccines and the ChAdOx1 nCoV-19 vaccine [24]. Both local (pain, swelling, and redness) and systemic (fever, malaise, fatigue, headache, and palpitation) ARs were more prevalent following the first vaccination dose than the second. However, they were short-lived, self-limiting symptoms and were mild or moderate in severity. They improved within 48 h, either spontaneously or with over-the-counter medications. No report of serious ARs either necessitated hospitalizations or induced disabilities or fatalities. These findings are consistent with those reported in other studies [10,25,26]. After two vaccination doses, younger age groups and females encountered more ARs, and reactogenicity was generally less frequent in the elderly. The increased likelihood of ARs at younger ages had been previously noticed by Kamal et al. 2021 [27], Ali et al. 2021 [28], Menni et al. 2021 [25], and Tequare et al. 2021 [29]. The potent immunoreactivity to vaccination observed in young subjects compared to the elderly increased the likelihood of ARs [10]. Consistent with previous studies [29,30], females were more prone to ARs due to the vigorous immune response provoked by the estrogen hormone [29,31]. It is worth mentioning that the salience of ARs following the first vaccine dose was a significant predictor of the production of anti-spike IgG antibodies, which was comparable to what had been reported by Jeong et al. 2021 [13]. Furthermore, the underlying comorbidity of the studied HCWs did not affect the severity of ARs to the vaccine.

The post-vaccine immune response incorporates several aspects, such as innate, humoral, and cellular immune responses. Although the humoral immune response represents only one aspect, it is far easier to detect serum antibody levels to assess the immune response to vaccines due to their widespread application and standardization [32,33]. In order to assess the long-term efficacy of the vaccine, the present study was designed to measure anti-spike IgG serum levels at three time points: (1) the start point just prior to the first vaccine dose (baseline), (2) the 12th w following the first vaccine dose (immediately prior to the second dose), and (3) the 24th w following the first vaccine dose (12 w following the second dose).

At the baseline point of the study, COVID-19 infection was reported in 54.1% of the HCWs; nevertheless, the anti-spike IgG was detectable in 35.6%, since it passed over six months in 71% of those who reported the previous infection. However, the seronegative HCWs (64.8%) showed seroconversion rates of 81.8% and 93.3% after the first and second vaccination doses, respectively. Of all the studied HCWs, 88.2% were seropositive following the first vaccination dose, while 95.7% were seropositive 12 w following the second dose, compared to a study conducted in England involving HCWs, who reported a 97.1% seropositivity rate after a single dose of the Oxford–AstraZeneca vaccine and before the second dose [34]. Meanwhile, a Korean study involving HCWs reported that the seropositivity rate after one dose of the Oxford–AstraZeneca vaccine was 68.2–100% after 66.2 days on average [35]. Furthermore, a study from Northern Ireland revealed that 86.9% of participants had been seropositive three weeks following the first vaccine dose, dropping to 74.7% instantly prior to the second dose. Overall, 99% of the participants were seropositive three weeks after the second dose, dropping to 90.5% six months after the first vaccine dose [36]. Accordingly, the variability in the seropositivity rates following the vaccine could be related to the different time points for collecting serum samples.

Our findings demonstrated that the Oxford–AstraZeneca vaccine successfully elicited a robust and sustained elevated anti-spike antibody response at 12th and 24th w compared to the baseline. These findings confirmed the vaccine’s efficacy discussed in the previous report on the safety and immunogenicity of the vaccine [26]. Moreover, minor variation between 12th and 24th w measurements of anti-spike IgG serum level coupled to the direct association between baseline and 12th and 24th w time points of anti-spike IgG serum level, particularly those with baseline antibody serum level exceeding the cut-off value, were significant findings in the study. In addition, a direct association between the 12th and 24th w measurement of anti-spike IgG serum level was evident. Overall, measuring basal antibody level before vaccination could predict antibody response after vaccination, and even more, measuring serum antibody level following the first vaccine dose could predict the response to the second dose. Younger HCWs demonstrated immunoreactivity to the vaccine despite the lack of correlation between age and anti-spike IgG serum level at the 12th- and 24th-week time points. Due to the known decline in T-cell-derived antibody production and B-lymphocyte generation that occurs with age, older individuals exhibit a weaker antibody response to various types of vaccines and a more rapid loss of antibodies [37]. Nevertheless, the non-significant correlation between age and anti-spike IgG serum level demonstrated in our work could be related to the under-representation of old age in the studied HCWs, as only 9.4% of the participants were aged between 60 and 80 years old.

Unlike mRNA vaccines (Pfizer-BioNTech and Moderna), which store the genetic instructions of building spike protein of SARS-CoV-2 in single-stranded RNA, the Oxford–AstraZeneca vaccine stores the instructions in double-stranded DNA [38]. Despite the extensive research on SARS-CoV-2 vaccines during the COVID-19 pandemic, the literature provides limited data on how they work and induce the immune system. Few reports have investigated mRNA vaccines and the expression of the spike protein. In their work, Cognetti and Miller (2021) reported that the spike protein enters the bloodstream and circulates for a week before complete clearance from circulation within one month [39]. However, regarding other types of vaccines, almost no data on the spike protein expression, concentration, half-life, and degradation are yet available. Therefore, detecting the spike protein serum level in recently vaccinated individuals and pursuing the post-vaccination antibody serum level could bring the complete picture of the successfulness of vaccine take and its dynamics to the spotlight.

Furthermore, among HCWs with a history of past COVID-19 infection before vaccination, a robust antibody response was observed at the 12th- and 24th-week time points compared to non-infected HCWs. Several studies confirmed that the decay of antibody titer was faster in vaccinated individuals who had never been infected than in those who had been infected prior to vaccination [34,36], which could be attributed to several factors. First, there is limited data on antibody persistence duration following COVID-19 infection or vaccination. Recent studies have detected antibodies circulating for seven months post-infection [40,41]. Additionally, another study on the Oxford–AstraZeneca vaccine demonstrated elevated antibody levels three months after a single dose [42]. Second, two recent studies proved that antibodies following COVID-19 originated in memory B cells and are likely necessary for long-term immunity [43,44], a mechanism by which most antiviral vaccines function [45]. Finally, vaccines might present viral proteins in slightly more conformations than the actual virus, resulting in variation in antigen and antibody kinetics [46]. Considering the immune response after infection is analogous to immune priming, the elevated antibody levels in individuals infected prior to vaccination most likely described the entirety of the antibodies developed following infection and vaccination [47].

In the present study, diabetic, hypertensive, and osteoarthritis patients demonstrated low immunoreactivity to the vaccine. Although COVID-19 vaccines were highly influential in general population cohorts, data on their efficacy amongst groups with distinct comorbidities were not satisfactory. Recent research on COVID-19 vaccines’ efficiency and immunogenicity amongst persons in clinical risk groups reported significantly frail seropositivity following a single dose of Oxford–AstraZeneca vaccine in diabetics [48]. Furthermore, reported seropositivity ensuing the vaccine’s first dose was 52.7% and 48% of patients with type 1 and type 2 diabetes. However, antibody levels were comparable after the second dose in patients with type 1 and type 2 diabetes and healthy controls. Several flaws in immunity were linked to diabetes, mainly cellular immunity; however, humoral immunity could also be affected [49]. The impaired humoral immune response had been previously reported with other vaccines, e.g., influenza and hepatitis B, in people with diabetes [50,51]. The association between osteoarthritis and diminished immunoreactivity to the vaccine can be attributed to old age or increased body mass index, which are major risk factors for osteoarthritis and have a negative impact on humoral immune response [52,53]. The dysregulation of cytokine profile associated with aging and obesity induces a low-grade inflammatory state that chronically activates the immune system and renders B cells refractory for further stimulation [54,55]. Consistent with Rifai et al. 2022 [56], we observed in hypertensive HCWs a lower trend in antibody production, and therefore, they might be more prone to have COVID-19 infections despite completing the vaccination schedule. Th2 subset number and IL-4 serum levels were significant reductions in hypertensive patients [57]. Otherwise, the hypertensive stimuli induce the Th1- and Th17-related cytokines, such as IFN-γ and IL-17A [58]. Therefore, the increased production of Th1 cytokines with suppression of Th2 cytokines triggered by angiotensin II contributed to suppressing humoral immunity and antibody production [59]. Moreover, the administration of angiotensin receptor blocker drugs somehow restored the balance of the Th subsets, hence improving the Th2 differentiation [60].

Before initiating the vaccination in the studied HCWs, confirmed past COVID-19 infections were reported in 54.1%, and more than two-thirds had encountered infections at least six months earlier. During the time spanning the scheduled two doses of the vaccine and the six-month follow-up period, the incidence of breakthrough COVID-19 cases among the studied HCWs had fallen to 6.4% and 23.5%, respectively. All reported breakthrough COVID-19 cases were mild, and none of the reported cases required hospitalization. As mentioned earlier, participants with past COVID-19 displayed significantly higher anti-spike IgG serum levels than those without, albeit the incidence of first and second breakthrough infections among those with past COVID-19 (8% and 24.6%, respectively) was surprisingly higher when compared to those without (5% and 22.2%, respectively). However, our findings are consistent with recent work during the Omicron Era [61]. Cerqueira-Silvae et al. (2022) reported that past COVID-19 infection offered robust protection against severe disease but not symptomatic infections, and this protection increased with hybrid immunity (combined past infection and vaccination) [61].

One limitation of the present study was the absence of sequencing data about the SARS-CoV-2 variants in the confirmed COVID-19 cases due to the limited resources restricting the sequencing process on a routine basis. It should be noted that Delta (B.1.617.2) and Omicron (B.1.1.529) variants were first reported in Egypt on August 24 and December 21, 2021, respectively, based on the WHO weekly epidemiologic updates [62], and started to circulate from then, which synchronized with our study and follow-up period. However, as described before [15], tracing the S gene expression status using TaqPath™ COVID-19 assay (Thermo Fischer Scientific) was an alternative route to identify SARS-CoV-2 variants [63]. A second limitation was the lack of a control arm; however, it was unattainable to recruit HCWs who could not be vaccinated and continue for nine months (the study duration) as a control arm without vaccination during the current COVID-19 pandemic. Hence, the design was a single-arm cohort study. Despite the adherence of the studied HCWs to infection, prevention, and control measures such as face masking, social distancing, and work restriction with proven COVID-19 infection, the relatively higher incidence of second breakthrough COVID-19 infection after the second dose of the vaccine (23.5%) compared to the first breakthrough (6.4%) can be accounted to several factors. A modest reduction in vaccine effectiveness against infection has been noticed with Delta variant [64], the predominated strain during the period between two doses of the vaccine (12 weeks). However, the multiple mutations in the receptor-binding domain of the spike protein in the Omicron variant might interfere with the performance of the currently available vaccines against it and empower its ability to spread among vaccinated individuals [63,65] widely. Furthermore, the easy and rapid dissemination of Omicron variants compared to Delta variants [66] and the longer follow-up period (24 weeks) after the second dose of vaccine could explain the increased incidence of breakthrough COVID-19 infection in the present study after the second dose. Nevertheless, antibody levels decayed over time after vaccination and remained detectable for more than six months following the first vaccination. These findings highlighted the significance of the vaccination and its substantial efficacy in alleviating the risk or severity of infections by new variants and the need for hospitalization. However, the waning of immunity over time, as evidenced in the current study by the increased incidence of infection in the Omicron era compared to the Delta era, necessitates further booster doses at regular intervals, particularly in high-risk groups [67]. Furthermore, the authors esteem the Egyptian public health authorities’ potential to facilitate access to primary and booster vaccine doses, despite the limited resources for mass vaccination. It reflects the successfulness of vaccination efforts and the application of infection, prevention, and control policies in Egyptian hospitals by Egyptian HCWs during the COVID-19 pandemic.

## 5. Conclusions

The Oxford–AstraZeneca vaccine is generally safe and is highly effective in preventing COVID-19 infections among Egyptian HCWs. It provides a robust, sustained humoral immune response successfully refractory to the constantly evolving SARS-CoV-2. Moreover, particular alertness should be directed to the elderly, males, and those who develop frail ARs following the first vaccine dose or those with associated comorbidities, particularly diabetes and hypertension. Finally, measuring serum anti-spike IgG levels before and after vaccination doses is a useful and standardized tool for predicting vaccine immunoreactivity and preparing for other alternatives in high-risk groups.

## Figures and Tables

**Table 1:** Baseline characteristics of the studied patients.

Variable	(*N* = 255)	%
**Age** (Years)		
Mean ± SD	40.7 ± 11.4	
Median (Min–Max)	38 (23–69)	
20–39	147	57.6
40–59	84	32.9
60–80	24	9.4
**Gender**		
Female	144	56.5
Male	111	43.5
**HCWs**		
Medical	183	71.8
Paramedical	72	28.2
**Associated comorbidity ^1^**	132	51.8
**Regular drug use ^2^**	57	22.4
**Ivermectin Administration**	138	54.1
Prophylactic	69	27.1
Therapeutic	15	5.8
Both	54	21.2
**Past COVID-19 infection**	138	54.1

^1^ Comorbidities included diabetes, hypertension, anemia, liver disease, neurologic disorders, thyroid disorders, osteoarthritis, chest disease, allergic rhinitis, urticaria, drug allergy, and food allergy. ^2^ Regular drugs included oral hypoglycemics, insulin, antihypertensives, nonsteroidal anti-inflammatory drugs, thyroid medication, and multivitamins.

**Table 2:** Factors associated with adverse reactions to vaccine.

	1st dose ARs			2nd dose AR		
Variable	Local *N* (%) 178 (69)	Systemic *N* (%) 212 (83.1)	*p* Value, OR (CI)	Local *N* (%) 120 (47.1)	Systemic *N* = 130 130 (51.0)	*p* Value, OR (CI)
**Age**						
20–39 *n* = 147	112 (62.9)	128 (60.4)	0.01 *, NA ^1^	84 (70.0)	72 (55.4)	0.001 *, NA ^1^
40–59 *n* = 84	54 (30.3)	69 (32.5)	0.01 *, NA ^2^	27 (22.5)	49 (37.7)	0.2, NA ^2^
60–80 *n* = 24	12 (6.7)	15 (7.1)		9 (7.5)	9 (6.9)	
**Sex**						
Female *n* = 144	116 (65.2)	128 (60.4)	<0.001 *, 5.2 (3.0–9.2) ^1^	84 (70.0)	75 (57.7)	<0.001 *, 2.9 (1.7–4.9) ^1^
Male *n* = 111	62 (34.8)	84 (39.6)	0.005 *, 2.6 (1.3–5.1) ^2^	36 (30.0)	55 (42.3)	0.7, 1.6 (0.7–1.8) ^2^
**Comorbidity**^3^ *n* = 132	90 (50.6)	112 (52.8)	0.6, 0.9 (0.5–1.5) ^1^ 0.5, 1.3 (0.7–2.5) ^2^	54 (45.0)	67 (51.5)	0.04 *, 0.6 (0.4–1.0) ^1^ 0.9, 1.0 (0.6–1.6) ^2^

AR; adverse reactions, OR; odds ratio, CI; confidence interval, NA; non-applicable. Data were analyzed by Pearson’s chi-square and Fisher’s exact tests when appropriate. * Significance; ^1^ Local adverse reactions; ^2^ Systemic adverse reactions; ^3^ Comorbidities included diabetes, hypertension, anemia, liver disease, neurologic disorders, thyroid disorders, osteoarthritis, chest disease, allergic rhinitis, urticaria, drug allergy, and food allergy.

**Table 3:** Immunoreactivity and seroconversion rate at different time points of the study.

Variable	(*N* = 255)	%
**SARS-CoV-2 IgG Immunoreactivity**		
Baseline	90	35.3
After 1st dose of vaccination	225	88.2
After 2nd dose of vaccination	244	95.7
**Seroconversion rate (*n* = 165)**		
After 1st dose of vaccination	135	81.8
After 2nd dose of vaccination	154	93.3

**Table 4:** Factors influencing Immunoreactivity to vaccine.

Variable	Immunoreactive *N* = 244 *N* (%)	Non-Immunoreactive *N* = 11 *N* (%)	*p*-Value	OR	CI
**Age**			<0.009 *	NA	NA
20–39 *n* = 147	144 (59.0)	3 (27.3)			
40–59 *n* = 84	80 (32.8)	4 (36.4)			
60–80 *n* = 24	20 (8.2)	4 (36.4)			
**Sex**			0.8	1.4	0.4–4.8
Female *n* = 144	137 (56.1)	7 (63.6)			
Male *n* = 111	107 (43.9)	4 (36.4)			
**HCWs**			0.5	0.7	0.2–2.4
Medical *n* = 183	176 (72.1)	7 (63.6)			
Paramedical *n* = 72	68 (27.9)	4 (36.4)			
**Ivermectin administration**					
Prophylaxis *n* = 123	120 (49.2)	3 (27.3)	0.2	0.4	0.1–1.5
Therapeutic *n* = 69	66 (27.0)	3 (27.3)	1.0	1.0	0.3–3.9
**Diabetes *n* = 28**	22 (9.0)	6 (54.5)	<0.001 *	12.1	3.4–42.9
**Hypertension *n* = 35**	31 (12.7)	4 (36.4)	0.049 *	3.9	1.1–14.2
**Anemia *n* = 18**	18 (7.4)	0 (0.0)	1.0	NA	NA
**Liver disease *n* = 7**	7 (2.9)	0 (0.0)	1.0	NA	NA
**Neurologic disorders *n* = 6**	6 (2.5)	0 (0.0)	1.0	NA	NA
**Thyroid disorders *n* = 13**	13 (5.3)	0 (0.0)	1.0	NA	NA
**Osteoarthritis *n* = 35**	28 (11.5)	7 (63.6)	<0.001 *	14.8	4.1–53.8
**Chest disease *n* = 12**	12 (4.9)	0 (0.0)	1.0	NA	NA
**Allergic rhinitis *n* = 46**	45 (18.4)	1 (9.1)	0.7	0.4	0.1–3.5
**Urticaria *n* = 19**	19 (7.8)	0 (0.0)	1.0	NA	NA
**Drug allergy *n* = 11**	11 (4.5)	0 (0.0)	1.0	NA	NA
**Food allergy *n* = 6**	6 (2.5)	0 (0.0)	1.0	NA	NA

OR; odds ratio, CI; confidence interval, NA; non-applicable. Data were analysed by Pearson’s chi-square and Fisher’s exact tests when appropriate. * Significance.

**Table 5:** Relation between adverse reactions and immunoreactivity to vaccine.

	Immunoreactivity to 1st Dose			Immunoreactivity to 2nd Dose		
Variable	Immuno-Reactive *N* = 225 *N* (%)	Non-Immunoreactive *N* = 30 *N* (%)	*p* Value, OR (CI)	Immuno-Reactive *N* = 244 *N* (%)	Non-Immunoreactive *N* = 11 *N* (%)	*p* Value, OR (CI)
**Local**	163 (72.4)	15 (50)	0.01 *, 2.6 (1.2–5.7)	114 (46.7)	6 (54.5)	0.6, 0.7 (0.2–2.5)
Pain	163 (72.4)	15 (50)	0.01 *, 2.6 (1.2–5.7)	122 (45.9)	3 (27.3)	0.4, 2.3 (0.6–8.7)
Swelling	42 (18.7)	6 (20.0)	0.9, 0.9 (0.4–2.4)	15 (6.1)	3 (27.3)	0.03 *, 0.2 (0.0–0.7)
Redness	38 (16.9)	3 (10.0)	0.4, 1.8 (0.5–6.3)	20 (8.2)	3 (27.3)	0.07, 0.2 (0.1–1.0)
Itching	10 (4.4)	0 (0.0)	0.6, NA	6 (2.5)	0 (0.0)	1.0, NA
**Systemic**	196 (87.1)	16 (53.3)	<0.001 *, 5.9 (2.6–13.4)	127 (52.0)	3 (27.3)	0.1, 2.9 (0.8–11.2)
Fever	138 (61.3)	9 (30.0)	0.001 *, 3.7 (1.6–8.5)	23 (9.4)	0 (0.0)	0.6, NA
Malaise	168 (61.3)	16 (53.3)	0.01 *, 2.6 (1.2–5.6)	95 (38.9)	3 (27.3)	0.5, 1.7 (4.4–6.6)
Fatigue	113 (50.2)	6 (20.0)	0.002 *, 4.0 (1.6–10.3)	52 (21.3)	0 (0.0)	0.1, NA
Headache	89 (39.6)	7 (23.3)	0.09, 2.2 (0.9–5.2)	36 (14.8)	3 (27.3)	0.3, 0.4 (0.1–1.8)
Nausea	10 (4.4)	0 (0.0)	0.6, NA	0 (0.0)	0 (0.0)	NA
Vomiting	7 (3.1)	0 (0.0)	1.0, NA	0 (0.0)	0 (0.0)	NA
Diarrhea	19 (8.4)	0 (0.0)	0.1, NA	0 (0.0)	0 (0.0)	NA
Dyspnea	12 (5.3)	0 (0.0)	0.4, NA	0 (0.0)	0 (0.0)	NA
Cough	7 (3.1)	0 (0.0)	1.0, NA	2 (0.8)	0 (0.0)	1.0, NA
Palpitation	10 (4.4)	3 (10.0)	0.2, 0.4 (0.1–1.6)	4 (1.6)	0 (0.0)	1.0, NA
Pruritis	3 (1.3)	0 (0.0)	1.0, NA	2 (0.8)	0 (0.0)	1.0, NA

OR; odds ratio, CI; confidence interval, NA; non-applicable. Data were analyzed by Pearson’s chi-square and Fisher’s exact tests when appropriate. * Significance.

**Table 6:** Relation between baseline Immunoreactivity and past COVID-19 infection.

Baseline Immunoreactivity	Past COVID-19 Infection		*p*	OR	CI
	Yes *N* = 138 *N* (%)	No *N* = 117 *N* (%)			
Immunoreactive	73 (52.9)	18 (15.4)	<0.001 *	6.2	3.4–11.3
Non-Immunoreactive	65 (39.1)	99 (84.6)			

OR; odds ratio, CI; confidence interval. Data were analyzed by Pearson’s chi-square test. * Significance.

## Data Availability

The data of this study are available from the corresponding author on reasonable request.
